# Melanoma Among Non-Hispanic Black Americans

**DOI:** 10.5888/pcd16.180640

**Published:** 2019-06-20

**Authors:** MaryBeth B. Culp, Natasha Buchanan Lunsford

**Affiliations:** 1 Surveillance and Health Services Research Program, American Cancer Society, Atlanta, Georgia; 2 Division of Cancer Prevention and Control, Centers for Disease Control and Prevention, Atlanta, Georgia

## Abstract

**Introduction:**

Few studies have examined melanoma incidence and survival rates among non-Hispanic black populations because melanoma risk is lower among this group than among non-Hispanic white populations. However, non-Hispanic black people are often diagnosed with melanoma at later stages, and the predominant histologic types of melanomas that occur in non-Hispanic black people have poorer survival rates than the most common types among non-Hispanic white people.

**Methods:**

We used the US Cancer Statistics 2001–2015 Public Use Research Database to examine melanoma incidence and 5-year survival among non-Hispanic black US populations.

**Results:**

From 2011 through 2015, the overall incidence of melanoma among non-Hispanic black people was 1.0 per 100,000, and incidence increased with age. Although 63.8% of melanomas in non-Hispanic black people were of unspecified histology, the most commonly diagnosed defined histologic type was acral lentiginous melanoma (16.7%). From 2001 through 2014, the relative 5-year melanoma survival rate among non-Hispanic black people was 66.2%.

**Conclusion:**

Although incidence of melanoma is relatively rare among non-Hispanic black populations, survival rates lag behind rates for non-Hispanic white populations. Improved public education is needed about incidence of acral lentiginous melanoma among non-Hispanic black people along with increased awareness among health care providers.

**Summary**

**What is already known on this topic?**

Non-Hispanic black people have lower incidence rates of melanoma than non-Hispanic white people; however, non-Hispanic black people are typically diagnosed at a later stage, have different proportions of histologic types of melanoma, and have poorer survival rates than non-Hispanic white people.

**What is added by this report?**

From 2011 through 2015, the histology of most melanomas diagnosed among non-Hispanic black patients was acral lentiginous melanoma. Five-year relative survival rates for acral lentiginous melanoma are lower than for the predominant histologic type diagnosed among non-Hispanic white people.

**What are the implications for public health practice?**

Improved education of health care providers about incidence of acral lentiginous melanoma among non-Hispanic black people is needed because of its atypical presentation and poor survival rates for this cancer.

## Introduction

Melanoma is one of the most common cancers in the United States, and incidence is increasing (1). Most melanomas are thought to be caused by a combination of exposure to ultraviolet radiation and characteristics of sun-sensitive skin (2). People with fair skin are generally at highest risk of melanoma; thus, recent research focused primarily on white and Hispanic populations (3). However, few studies of melanoma were conducted among non-Hispanic black populations (4). One of these studies found that ultraviolet radiation is associated with skin cancer risk among black men (5).

Skin type or complexion is a key risk factor for skin cancer (6). Melanin, which is produced in the skin, gives skin, hair, and eyes their color and protects the deep layers of skin cells from ultraviolet radiation damage (6). Skin that produces more melanin is naturally darker and provides more sun protection from ultraviolet radiation than light skin, which produces less melanin (6). Although exposure to ultraviolet radiation can cause DNA damage to all types of skin, some melanoma histology types are not attributable to exposure (6,7). Although black men and women in the United States are at lower risk of melanoma than white men and women, they often have melanoma diagnosed at a later stage and have poorer survival as a result (4,8). Furthermore, a range of skin complexions among non-Hispanic black people leads to variable risk from ultraviolet radiation exposure (4).

Previous analyses found that melanoma histologic type and the body site where it occurs differ by race (4,9). Acral lentiginous melanoma (ALM), which typically presents on the palms of hands, soles of feet, or nail beds, is associated with poor survival rates, and a greater proportion of melanomas diagnosed among non-Hispanic black people are ALM than are melanomas diagnosed among non-Hispanic whites (10,11). We examined melanoma incidence and survival among non-Hispanic black populations in the United States by age, stage at diagnosis, anatomic site, and histology.

## Methods

We examined population-based cancer registry incidence data from the US Cancer Statistics (USCS) 2001–2015 Public Use Research Database (12). This database combines data from the Centers for Disease Control and Prevention’s National Program of Cancer Registries (NPCR) and from the National Cancer Institute’s Surveillance, Epidemiology, and End Results (SEER) program, covering the entire US population for 2011 through 2015. This data set excludes cancer diagnoses identified only on autopsy or death certificate and cancers in patients of unknown age or sex. Cancers were coded by using the *International Classification of Diseases for Oncology* current at the time of diagnosis and later converted to codes in the *International Classification of Diseases for Oncology*, third edition (ICD-O-3) (13). We defined invasive melanomas of the skin as having an ICD-O-3 site code of C440–C449 and an ICD-O-3 histology code of 8720–8790; analyses of histology data were limited to microscopically confirmed cases, and we excluded lentigo maligna melanoma histology because of its low incidence among non-Hispanic black populations. We limited our research to non-Hispanic black populations, except in some instances when non-Hispanic white populations were used as a comparison group. Non-Hispanic black and non-Hispanic white race/ethnicity was identified by the demographic information in the cancer medical case reports that were submitted to state cancer registries. We also presented data for the US Census region in which patients lived at the time of diagnosis: Northeast, Midwest, South, or West.

We used SEER*Stat software version 8.3.5 (National Cancer Institute) to calculate age-adjusted rates per 100,000 population (based on the 2000 US standard population), rate ratios, 95% confidence intervals, average annual counts, and 5-year relative survival. Rates based on a count of fewer than 16 patients were not reported because of concerns for rate stability (14). We limited our analyses to the most recent 5 years of data available to present the most current incidence rates. Rate ratios were considered to be significant if they differed from 1 at *P* < .05. Survival data were taken from NPCR registries of the 39 participating states with survival data, which covered 81.1% of the US population for 2001 through 2014 (15). Survival rates were calculated by the actuarial method (16). The data used in this study were publicly available and contained no patient identifiable information; therefore, no institutional review board oversight was needed.

## Results

From 2011 through 2015, 1,795 non-Hispanic black people were diagnosed with melanomas in the United States, an average of 359 per year (incidence rate, 1.0 per 100,000) (Table 1). Rates were similar for men and women except in the oldest age group (65 or older), and increased with age, with the highest rates among men aged 65 or older (5.5 per 100,000). Half (55.3%) of all melanomas were diagnosed at a localized stage. Women were slightly more likely to be diagnosed at a localized stage (56.7%) than men (53.6%). Lower extremities, including legs and feet, were the most common anatomic sites where melanoma occurred among non-Hispanic black people (48.2%). Patterns by anatomic site did not vary widely by sex, although a slightly higher percentage of women than men had melanoma diagnosed on lower extremities, and men had slightly higher percentages of melanoma diagnosed on the head and neck and trunk than women. Most melanomas had nonspecific histology (63.8%). Among melanomas with specific histology, ALM were the most common (16.7%). Finally, more than half (58.7%) of melanoma diagnoses were among people in the southern United States compared with the Northeast (17.3%), Midwest (13.7%), and West (10.3%).

**Table 1:** Age-Adjusted Melanoma Incidence Rates Among Non-Hispanic Black People, United States, 2011–2015^a^

Variable	All			Male			Female		
	Rate per 100,000 (95% CI)	AAC^b^	N (%)	Rate per 100,000 (95% CI)	AAC^b^	N (%)	Rate per 100,000 (95 % CI)	AAC^b^	N (%)
**Total**	1.0 (1.0–1.1)	359	1,795 (100.0)	1.2 (1.1–1.2)	169	843 (100.0)	1.0 (0.9–1.0)	190	952 (100.0)
**Age, y**									
0–44^c^	0.2 (0.2–0.3)	56	281 (15.7)	0.2 (0.2–0.2)	24	118 (14.0)	0.3 (0.2–0.3)	33	163 (17.1)
45–64	1.3^c^ (1.2–1.4)	131	653 (36.4)	1.4^c^ (1.2–1.5)	66	329 (39.0)	1.2^c^ (1.1–1.3)	65	324 (34.0)
≥65	4.6^c^ (4.3–4.9)	172	861 (48.0)	5.5^c^ (4.9–6.1)	79	396 (47.0)	4.1^c^ (3.7–4.4)	93	465 (48.8)
**Stage at diagnosis**									
Localized	0.6 (0.5–0.6)	198	992 (55.3)	0.6 (0.6–0.7)	90	452 (53.6)	0.5 (0.5–0.6)	108	540 (56.7)
Regional	0.2^c^ (0.2–0.2)	65	327 (18.2)	0.2^c^ (0.2–0.2)	33	167 (19.8)	0.2^c^ (0.1–0.2)	32	160 (16.8)
Distant	0.2^c^ (0.1–0.2)	59	296 (16.5)	0.2^c^ (0.2–0.2)	29	144 (17.1)	0.2^c^ (0.1–0.2)	30	152 (16.0)
Unstaged	0.1^c^ (0.1–0.1)	36	180 (10.0)	0.1^c^ (0.1–0.1)	18	80 (9.5)	0.1^c^ (0.1–0.1)	22	100 (10.5)
**Anatomic site**									
Head and neck^c^	0.1 (0.1–0.1)	34	166 (9.2)	0.1 (0.1–0.2)	18	87 (10.3)	0.1 (0.1–0.1)	16	79 (8.3)
Trunk	0.1^c^ (0.1–0.2)	53	263 (14.7)	0.2^c^ (0.2–0.2)	30	149 (17.7)	0.1^c^ (0.1–0.1)	23	114 (12.0)
Upper extremity	0.2^c^ (0.1–0.2)	56	278 (15.5)	0.2^c^ (0.2–0.2)	28	140 (16.6)	0.1^c^ (0.1–0.2)	28	138 (14.5)
Lower extremity	0.5^c^ (0.5–0.5)	173	865 (48.2)	0.5^c^ (0.4–0.5)	72	362 (42.9)	0.5^c^ (0.5–0.6)	101	503 (52.8)
NOS and other	0.1^c^ (0.1–0.1)	51	223 (12.4)	0.1 (0.1–0.2)	24	105 (12.5)	0.1^c^ (0.1–0.1)	27	118 (12.4)
**Histology^d^**									
Superficial spreading melanoma	0.1 (0.1–0.1)	37	183 (10.4)	0.1 (0.1–0.1)	17	79 (9.6)	0.1 (0.1–0.1)	21	104 (11.1)
Nodular melanoma	0.1 (0.1–0.1)	32	161 (9.1)	0.1 (0.1–0.1)	20	75 (9.1)	0.1 (0.1–0.1)	20	86 (9.2)
Acral lentiginous melanoma	0.2^c^ (0.2–0.2)	59	294 (16.7)	0.2^c^ (0.2–0.2)	28	139 (16.8)	0.2^c^ (0.1–0.2)	31	155 (16.6)
Melanoma NOS and other	0.6^c^ (0.6–0.7)	225	1,124 (63.8)	0.7^c^ (0.7–0.8)	107	533 (64.5)	0.6^c^ (0.5–0.6)	118	591 (63.1)
**US Census region**									
Northeast	1.0 (0.9–1.1)	62	310 (17.3)	1.1 (0.9–1.3)	27	136 (16.1)	1.0 (0.8–1.1)	35	174 (18.3)
Midwest	0.8^c^ (0.7–0.9)	49	246 (13.7)	0.8^c^ (0.7–1.0)	23	106 (12.6)	0.8^c^ (0.6–0.9)	28	140 (14.7)
South	1.1 (1.0–1.1)	211	1,054 (58.7)	1.2 (1.1–1.3)	101	504 (59.8)	1.0 (0.9–1.1)	110	550 (57.8)
West	1.2 (1.0–1.4)	37	185 (10.3)	1.3 (1.1–1.7)	22	97 (11.5)	1.0 (0.8–1.3)	19	88 (9.2)

Abbreviations: AAC, average annual counts; CI, confidence interval; NOS, not otherwise specified.

a Source: National Program of Cancer Registries and Surveillance, Epidemiology, and End Results SEER*Stat database (12). Rates are age-adjusted to the 2000 US standard population (19 age groups – Census P25-1130).

b Average annual counts may not sum to total because of rounding.

c This rate is significantly different (*P* < .05) from the rate for the reference group (first listed group) as determined by rate ratios (not shown).

d Histologic analysis was limited to microscopically confirmed cases. Lentigo maligna melanoma histology was excluded because of low numbers.

**Figure 1:**
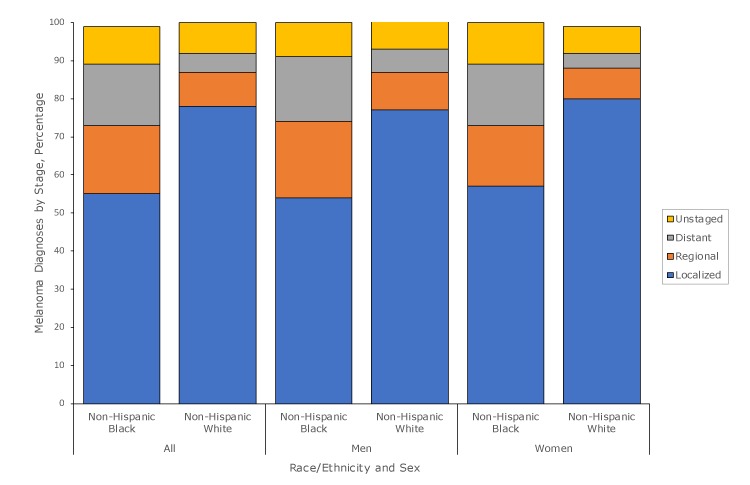
Percentage of non-Hispanic black and non-Hispanic white population diagnosed with melanoma, by stage at diagnosis, United States, 2011–2015.

Half (55%) of non-Hispanic black people and 78% of non-Hispanic white people were diagnosed with melanoma at a localized stage; 18% of non-Hispanic black people and 9% of non-Hispanic white people were diagnosed with regional-stage melanoma (Figure 1). More non-Hispanic black people were diagnosed with melanoma with distant metastasis (16%) than non-Hispanic white people (5%) or with unstaged melanoma (10%) than non-Hispanic white people (8%). Furthermore, fewer non-Hispanic black people than non-Hispanic white people were diagnosed with superficial spreading melanoma (SSM) (non-Hispanic black, 29%; non-Hispanic white, 79%), whereas the proportions diagnosed with nodular melanoma were similar (non-Hispanic black, 25%; non-Hispanic white, 19%). Although rates for ALM among non-Hispanic black people and non-Hispanic white people were similar (0.2 per 100,000), the proportion of ALM diagnosed among non-Hispanic black people was much higher (non-Hispanic black, 46% vs non-Hispanic white, 2%).

**Table:** 

Stage	All		Men		Women	
	Non-Hispanic Black	Non-Hispanic White	Non-Hispanic Black	Non-Hispanic White	Non-Hispanic Black	Non-Hispanic White
Localized	55	78	54	77	57	80
Regional	18	9	20	10	17	8
Distant	16	5	17	6	16	4
Unstaged	10	8	9	8	11	7

We examined data on 2,848 melanomas diagnosed from 2001 through 2014 (Table 2). Overall, the relative 5-year melanoma survival rate among non-Hispanic black populations was 66.2%, compared with 90.1% for non-Hispanic white populations. Survival decreased with age and was poorer among men (Table 2). The earlier the stage at diagnosis, the higher the survival rate (85.8% for localized melanoma compared with 52.8% for melanoma with regional spread and 19.0% for melanoma with distant metastasis). By anatomic site, melanomas diagnosed on the upper extremities had the best survival rates (82.2%), whereas those on the lower extremities had the poorest survival rates (67.6%). Melanomas with unknown anatomic site (not otherwise specified and other) had a poor relative survival rate (26.2%). Survival rates were highest among people diagnosed with SSM (91.1%), whereas rates were poorest among those diagnosed with nodular melanoma (56.6%) and ALM (66.1%).

**Table 2:** Relative 5-Year Survival, Melanoma Among Non-Hispanic Black People, National Program of Cancer Registries, 2001–2014^a^

Variable	All		Male		Female	
	N (%)	Relative Survival (95% CI)	N (%)	Relative Survival (95% CI)	N (%)	Relative Survival (95% CI)
**All ages**	2,848 (100.0)	66.2% (63.8–68.5%)	1,202 (100.0)	59.2% (55.3–62.9%)	1,646 (100.0)	71.1% (68.1–73.9%)
**Age, y**						
0–44	579 (20.3)	75.9% (71.8–79.5%)	227 (18.9)	64.1% (56.5–70.7%)	352 (21.4)	83.0% (78.3–86.8%)
45–64	1,069 (37.5)	68.2% (64.6–71.4%)	503 (41.8)	59.0% (53.5–64.0%)	566 (34.4)	76.2% (71.5–80.2%)
≥65	1,200 (42.1)	58.7% (54.3–62.9%)	472 (39.3)	56.6% (49.1–63.3%)	728 (44.2)	60.1% (54.4–65.2%)
**Stage at diagnosis**						
Localized	1,484 (52.1)	85.8% (82.6–88.5%)	571 (47.5)	81.5% (75.5–86.2%)	913 (55.5)	88.4% (84.3–91.4%)
Regional	596 (20.9)	52.8% (47.6–57.8%)	265 (22.0)	46.4% (38.6–53.8%)	331 (20.1)	57.7% (50.6–64.1%)
Distant	444 (15.6)	19.0% (14.8–23.7%)	219 (18.2)	15.0% (9.8–21.1%)	225 (13.7)	22.3% (16.1–29.2%)
Unstaged	324 (11.4)	63.2% (55.9–69.7%)	147 (12.2)	57.4% (46.0–67.2%)	177 (10.8)	67.2% (58.2–74.7%)
**Anatomic site**						
Head and neck	296 (10.4)	69.7% (62.2–76.1%)	153 (12.7)	62.2% (51.2–71.5%)	143 (8.7)	77.6% (66.7–85.3%)
Trunk	413 (14.5)	77.2% (71.2–82.1%)	200 (16.6)	76.0% (66.4–83.2%)	213 (12.9)	77.8% (69.7–84.0%)
Upper extremity	380 (13.3)	82.2% (75.8–87.1%)	161 (13.4)	81.5% (70.5–88.7%)	219 (13.3)	82.9% (74.3–88.8%)
Lower extremity	1,413 (49.6)	67.6% (64.0–70.9%)	532 (44.3)	57.2% (51.1–62.9%)	881 (53.5)	73.5% (69.1–77.3%)
NOS and other	346 (12.1)	26.2% (20.9–31.8%)	156 (13.0)	16.3% (10.2–23.7%)	190 (11.5)	33.9% (26.1–41.9%)
**Histology^b^**						
Superficial spreading melanoma	321 (11.4)	91.1% (83.9–95.2%)	134 (11.3)	92.7% (75.8–98.0%)	187 (11.5)	90.0% (81.1–94.9%)
Nodular melanoma	236 (8.4)	56.6% (47.8–64.5%)	105 (8.9)	49.9% (37.2–61.4%)	131 (8.1)	62.3% (50.0–72.5%)
Acral lentiginous melanoma	452 (16.1)	66.1% (59.2–72.1%)	174 (14.7)	54.7% (43.0–65.0%)	278 (17.1)	72.6% (64.0–79.5%)
Melanoma NOS and other	1,801 (64.1)	62.1% (59.1–64.9%)	770 (65.1)	54.2% (49.5–58.7%)	1,031 (63.4)	67.6% (63.8–71.1%)

Abbreviations: CI, confidence interval; NOS, not otherwise specified.

a Relative 5-year survival refers to the percentage of people with diagnosed melanoma alive 5 years following diagnosis compared with the general population. Source: National Program of Cancer Registries SEER*Stat database (15).

b Histologic analysis was limited to microscopically confirmed cases. Lentigo maligna melanoma histology was excluded because of low case counts.

**Figure 2:**
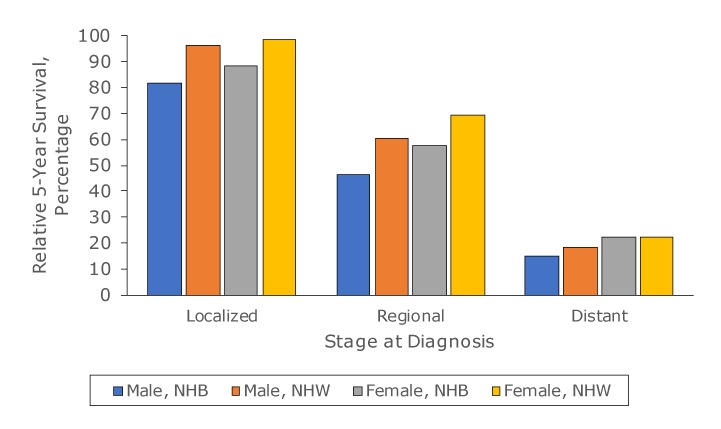
Five-year relative survival rate of melanoma (percentage of people diagnosed with melanoma alive 5 years following diagnosis) among non-Hispanic black (NHB) and non-Hispanic white (NHW) populations, by stage at diagnosis, United States, 2011–2015.

Relative survival rates differed between non-Hispanic black populations and non-Hispanic white populations by sex and by stage at diagnosis (Figure 2). Overall, regardless of stage at diagnosis, survival was lower among non-Hispanic black people than among non-Hispanic white people. Survival for localized stage for non-Hispanic black populations was 85.8% versus 97.5% for non-Hispanic white people; for regional stage, survival was 52.8% for non-Hispanic black people versus 63.8% for non-Hispanic white people and for distant stage, 19.0% for non-Hispanic black people versus 19.8% for non-Hispanic white people. Differences by sex showed similar patterns across all stages at diagnosis, with non-Hispanic black men and women having lower survival rates than non-Hispanic white men and women.

**Table:** 

Sex	Race	Stage at Diagnosis		
		Localized	Regional	Distant
Male	NHB	81.5	46.4	15.0
	NHW	96.6	60.3	18.4
Female	NHB	88.4	57.7	22.3
	NHW	98.4	69.3	22.6

## Discussion

Our analysis of survival data from 39 states (81.1% of the US population) showed lower survival rates among non-Hispanic black people with melanoma than among non-Hispanic white people (8,17,18). Our results are consistent with previously published studies of non-Hispanic black populations that showed melanoma incidence rates increasing with age (4). A smaller proportion of non-Hispanic black people were diagnosed with melanoma in localized stages than with melanoma that had spread to distant anatomic sites, as shown in a previous study in Georgia (19). Delays in diagnosis of disease may lead to low survival rates; therefore, these differences highlight the need for increased awareness of melanoma in non-Hispanic black people among both the public and health care providers and more timely diagnosis.

Previous research indicated that most melanomas among non-Hispanic black people occur on the lower limbs and hip or lower extremities, including soles of the feet (4,9). One study found that ALM was located on the lower limbs 78.3% of the time (10). Our results were consistent with these findings, showing that most melanomas among non-Hispanic black women were diagnosed on the lower extremities (4). Furthermore, lower-extremity melanomas had the worst survival by anatomic site overall among both sexes. Differences by sex in melanoma incidence and anatomic site may indicate differences in use of sun protection, because non-Hispanic black women report more frequent use of sun protection than do non-Hispanic black men (20,21).

Dark skin confers more protection against ultraviolet radiation than fair skin, but skin type and skin tone can vary widely among people of the same race/ethnicity (22). About 13% of non-Hispanic black people reported getting a sunburn in the past 12 months; of these, 19% reported getting 4 or more sunburns in the same period (23). Many non-Hispanic black people perceive themselves to be at lower risk of skin cancer and report using sun protection less frequently than non-Hispanic white people (20,21,24,25). However, educational interventions have shown promise in improving knowledge and use of sun protection among people with dark skin (26).

ALM was the most common melanoma subtype diagnosed among non-Hispanic black populations in our study. Survival is poorer for ALM than for SSM, the most common subtype diagnosed among non-Hispanic white populations. Other studies have found that ALM is associated with lower survival rates than other subtypes and that the high proportion of ALM among non-Hispanic black people may be partially responsible for poor survival outcomes (8,10,27). Unlike other types of melanoma, ALM may not be related to ultraviolet radiation, which may account for its higher incidence among non-Hispanic black populations than among non-Hispanic white populations (4,7). One study indicated that risk factors for ALM include previous trauma or nevi (a type of mole) on soles or toes and possible genetic or environmental factors (7). More research is warranted into the causes of the ALM subtype, which affects a high proportion of non-Hispanic black people with melanoma.

Survival was also poor among non-Hispanic black people for nodular melanoma. As with ALM, nodular melanoma is associated with poor survival and is often diagnosed in advanced stages (9,28). Both ALM and nodular melanoma have atypical presentation, not adhering to the “ABCD” (asymmetry, border, color, diameter) guidelines for skin mole examination traditionally used to identify melanoma (29). ALM often resembles a bruise or lesion on the hand or foot or a bruised fingernail; nodular melanomas are often round and symmetrical with a single color (30). One study suggested that the atypical location of plantar melanomas is what leads to the delay in diagnosis so that patients present with deep tumors at late stages that are hard to treat successfully (31).

Our study had limitations. One limitation was the exclusion from the incidence database of people with melanomas reported only on autopsy or death certificates; however, these patients constitute less than 5% of the database and therefore would not affect the incidence rates significantly if they were included (12). Another limitation is that the survival database does not cover the entire US population; however, the 81.1% of the population included was the best representation of survival rates available.

A strength of our analysis is its comprehensive coverage of USCS data, which contain cancer incidence rates for the entire US population. Furthermore, this study provides an in-depth look at melanoma incidence and survival rates among the non-Hispanic black population that have not been previously reported.

Increased awareness is needed among the public and the medical community of the prevalence of the ALM histology type and the common anatomic sites where it occurs because of ALM’s atypical presentation. Opportunities exist to provide increased education and behavioral counseling among non-Hispanic black populations about minimizing exposure to ultraviolet radiation to reduce skin cancer risk and about the need for regular skin checks by medical professionals, particularly in non-sun–exposed areas such as the feet (32).

## References

[1] US Cancer Statistics Working Group. United States cancer statistics: 1999–2014 incidence and mortality web-based report. Atlanta (GA): US Department of Health and Human Services, Centers for Disease Control and Prevention and National Cancer Institute; 2017. www.cdc.gov/uscs. Accessed June 13, 2018.

[2] Armstrong BK , Kricker A . The epidemiology of UV induced skin cancer. J Photochem Photobiol B 2001;63(1-3):8–18. 10.1016/S1011-1344(01)00198-1 11684447

[3] Richards TB , Johnson CJ , Tatalovich Z , Cockburn M , Eide MJ , Henry KA , Association between cutaneous melanoma incidence rates among white US residents and county-level estimates of solar ultraviolet exposure. J Am Acad Dermatol 2011;65(5, Suppl 1):S50–7. 10.1016/j.jaad.2011.04.035 22018067 PMC3206292

[4] Myles ZM , Buchanan N , King JB , Singh S , White A , Wu M , Anatomic distribution of malignant melanoma on the non-Hispanic black patient, 1998–2007. Arch Dermatol 2012;148(7):797–801. 10.1001/archdermatol.2011.3227 22801611

[5] Hu S , Ma F , Collado-Mesa F , Kirsner RS . UV radiation, latitude, and melanoma in US Hispanics and blacks. Arch Dermatol 2004;140(7):819–24. 10.1001/archderm.140.7.819 15262692

[6] Tadokoro T , Kobayashi N , Zmudzka BZ , Ito S , Wakamatsu K , Yamaguchi Y , UV-induced DNA damage and melanin content in human skin differing in racial/ethnic origin. FASEB J 2003;17(9):1177–9. 10.1096/fj.02-0865fje 12692083

[7] Durbec F , Martin L , Derancourt C , Grange F . Melanoma of the hand and foot: epidemiological, prognostic and genetic features. A systematic review. Br J Dermatol 2012;166(4):727–39. 10.1111/j.1365-2133.2011.10772.x 22175696

[8] Pollack LA , Li J , Berkowitz Z , Weir HK , Wu XC , Ajani UA , Melanoma survival in the United States, 1992 to 2005. J Am Acad Dermatol 2011;65(5, Suppl 1):S78–86. 10.1016/j.jaad.2011.05.030 22018071 PMC4890628

[9] Mahendraraj K , Sidhu K , Lau CS , McRoy GJ , Chamberlain RS , Smith FO . Malignant melanoma in African-Americans: a population-based clinical outcomes study involving 1,106 African-American patients from the Surveillance, Epidemiology, and End Result (SEER) database (1988–2011). Medicine (Baltimore) 2017;96(15):e6258. 10.1097/MD.0000000000006258 28403068 PMC5403065

[10] Bradford PT , Goldstein AM , McMaster ML , Tucker MA . Acral lentiginous melanoma: incidence and survival patterns in the United States, 1986–2005. Arch Dermatol 2009;145(4):427–34. 10.1001/archdermatol.2008.609 19380664 PMC2735055

[11] Cormier JN , Xing Y , Ding M , Lee JE , Mansfield PF , Gershenwald JE , Ethnic differences among patients with cutaneous melanoma. Arch Intern Med 2006;166(17):1907–14. 10.1001/archinte.166.17.1907 17000949

[12] SEER*Stat Database: NPCR and SEER Incidence — U.S. cancer statistics public use database, Nov 2017 submission (2001–2015). Atlanta (GA): US Department of Health and Human Services, Centers for Disease Control and Prevention and National Cancer Institute; 2017. https://www.cdc.gov/cancer/uscs/public-use/index.htm. Accessed June 13, 2018.

[13] World Health Organization. (‎2013)‎. International classification of diseases for oncology (‎‎ICD-O)‎‎ third edition, first revision, third edition. http://www.who.int/iris/handle/10665/96612. Accessed June 13, 2018.

[14] National Program of Cancer Registries and Surveillance, Epidemiology, and End Results SEER*Stat Database: NPCR and SEER incidence — US cancer statistics 2001–2015 public use research database data standards and data dictionary. Atlanta (GA): US Department of Health and Human Services, Centers for Disease Control and Prevention and National Cancer Institute; 2017. https://www.cdc.gov/cancer/uscs/public-use/pdf/data-dictionary.pdf. Accessed June 13, 2018.

[15] National Program of Cancer Registries SEER*Stat Database: NPCR survival analytic file 2001–2014. US Department of Health and Human Services, Centers for Disease Control and Prevention. Released July 2018, based on the November 2017 submission. Accessed June 13, 2018.

[16] Cutler SJ , Ederer F . Maximum utilization of the life table method in analyzing survival. J Chronic Dis 1958;8(6):699–712. 10.1016/0021-9681(58)90126-7 13598782

[17] Wang Y , Zhao Y , Ma S . Racial differences in six major subtypes of melanoma: descriptive epidemiology. BMC Cancer 2016;16(1):691. 10.1186/s12885-016-2747-6 27576582 PMC5004333

[18] Ward-Peterson M , Acuña JM , Alkhalifah MK , Nasiri AM , Al-Akeel ES , Alkhaldi TM , Association between race/ethnicity and survival of melanoma patients in the United States over 3 decades: a secondary analysis of SEER data. Medicine (Baltimore) 2016;95(17):e3315. 10.1097/MD.0000000000003315 27124020 PMC4998683

[19] Culp M , Wagner Robb S , Bayakly AR , Vena JE . Racial and socio-economic disparities in melanoma incidence rates in Georgia: 2000–2011. J Ga Public Health Assoc 2015;5(2):140–8.

[20] Holman DM , Berkowitz Z , Guy GP Jr , Hawkins NA , Saraiya M , Watson M . Patterns of sunscreen use on the face and other exposed skin among US adults. J Am Acad Dermatol 2015;73(1):83–92.e1. 10.1016/j.jaad.2015.02.1112 26002066 PMC4475428

[21] Buchanan Lunsford N , Berktold J , Holman DM , Stein K , Prempeh A , Yerkes A . Skin cancer knowledge, awareness, beliefs and preventive behaviors among black and Hispanic men and women. Prev Med Rep 2018;12:203–9. 10.1016/j.pmedr.2018.09.017 30364862 PMC6199782

[22] Pichon LC , Landrine H , Corral I , Hao Y , Mayer JA , Hoerster KD . Measuring skin cancer risk in African Americans: is the Fitzpatrick Skin Type Classification Scale culturally sensitive? Ethn Dis 2010;20(2):174–9. 20503899

[23] Holman DM , Berkowitz Z , Guy GP Jr , Hartman AM , Perna FM . The association between demographic and behavioral characteristics and sunburn among U.S. adults — National Health Interview Survey, 2010. Prev Med 2014;63:6–12. 10.1016/j.ypmed.2014.02.018 24589442 PMC4535173

[24] Buster KJ , You Z , Fouad M , Elmets C . Skin cancer risk perceptions: a comparison across ethnicity, age, education, gender, and income. J Am Acad Dermatol 2012;66(5):771–9. 10.1016/j.jaad.2011.05.021 21875760 PMC3766358

[25] Agbai ON , Buster K , Sanchez M , Hernandez C , Kundu RV , Chiu M , Skin cancer and photoprotection in people of color: a review and recommendations for physicians and the public. J Am Acad Dermatol 2014;70(4):748–62. 10.1016/j.jaad.2013.11.038 24485530

[26] Kailas A , Botwin AL , Pritchett EN , Jackson-Richards D , Lewis S , Sadhwani D , Assessing the effectiveness of knowledge-based interventions in increasing skin cancer awareness, knowledge, and protective behaviors in skin of color populations. Cutis 2017;100(4):235–40. 29136057

[27] Wu XC , Eide MJ , King J , Saraiya M , Huang Y , Wiggins C , Racial and ethnic variations in incidence and survival of cutaneous melanoma in the United States, 1999–2006. J Am Acad Dermatol 2011;65(5, Suppl 1):S26–37. 10.1016/j.jaad.2011.05.034 22018064

[28] Shaikh WR , Dusza SW , Weinstock MA , Oliveria SA , Geller AC , Halpern AC . Melanoma thickness and survival trends in the United States, 1989 to 2009. J Natl Cancer Inst 2015;108(1):djv294. 26563354 10.1093/jnci/djv294 PMC4857148

[29] Kalkhoran S , Milne O , Zalaudek I , Puig S , Malvehy J , Kelly JW , Historical, clinical, and dermoscopic characteristics of thin nodular melanoma. Arch Dermatol 2010;146(3):311–8. 10.1001/archdermatol.2009.369 20231503

[30] Chamberlain AJ , Fritschi L , Kelly JW . Nodular melanoma: patients’ perceptions of presenting features and implications for earlier detection. J Am Acad Dermatol 2003;48(5):694–701. 10.1067/mjd.2003.216 12734497

[31] Franke W , Neumann NJ , Ruzicka T , Schulte KW . Plantar malignant melanoma — a challenge for early recognition. Melanoma Res 2000;10(6):571–6. 10.1097/00008390-200012000-00009 11198479

[32] US Preventive Services Task Force. Screening for skin cancer: US Preventive Services Task Force recommendation statement. Ann Intern Med 2009;150(3):188–93. 10.7326/0003-4819-150-3-200902030-00008 19189908

